# Rhizopine biosensors for plant‐dependent control of bacterial gene expression

**DOI:** 10.1111/1462-2920.16288

**Published:** 2022-12-04

**Authors:** Timothy L. Haskett, Barney A. Geddes, Ponraj Paramasivan, Patrick Green, Samir Chitnavis, Marta D. Mendes, Beatriz Jorrín, Hayley E. Knights, Tahlia R. Bastholm, Joshua P. Ramsay, Giles E. D. Oldroyd, Philip S. Poole

**Affiliations:** ^1^ Department of Plant Sciences University of Oxford Oxford UK; ^2^ Department of Microbiological Sciences North Dakota State University Fargo North Dakota USA; ^3^ Sainsbury Laboratory University of Cambridge Cambridge UK; ^4^ Curtin Medical School and Curtin Health Innovation Research Institute Curtin University Perth Western Australia Australia; ^5^ Crop Science Centre University of Cambridge Cambridge UK

## Abstract

Engineering signalling between plants and microbes could be exploited to establish host‐specificity between plant‐growth‐promoting bacteria and target crops in the environment. We previously engineered rhizopine‐signalling circuitry facilitating exclusive signalling between rhizopine‐producing (*RhiP*) plants and model bacterial strains. Here, we conduct an in‐depth analysis of rhizopine‐inducible expression in bacteria. We characterize two rhizopine‐inducible promoters and explore the bacterial host‐range of rhizopine biosensor plasmids. By tuning the expression of rhizopine uptake genes, we also construct a new biosensor plasmid pSIR05 that has minimal impact on host cell growth in vitro and exhibits markedly improved stability of expression in situ on *RhiP* barley roots compared to the previously described biosensor plasmid pSIR02. We demonstrate that a sub‐population of *Azorhizobium caulinodans* cells carrying pSIR05 can sense rhizopine and activate gene expression when colonizing *RhiP* barley roots. However, these bacteria were mildly defective for colonization of *RhiP* barley roots compared to the wild‐type parent strain. This work provides advancement towards establishing more robust plant‐dependent control of bacterial gene expression and highlights the key challenges remaining to achieve this goal.

## INTRODUCTION

Plant roots are colonized by a plethora of microorganisms that adopt a range of life strategies spanning the continuum from pathogenic to beneficial (Bai et al., 2022; Knights et al., 2021). As such, the root microbiome is critical to plant health and has become a prime target for sustainably maintaining crop productivity (Kaul et al., 2021). Bacteria, representing the largest fraction of root microbiomes (Bulgarelli et al., 2013; Tkacz et al., 2020), promote plant growth by several mechanisms including nitrogen fixation, phosphorus solubilization, growth hormone biosynthesis, antimicrobial production and deterrence of pathogens (Glick, 2012; Souza et al., 2015). While some of these ‘plant growth‐promoting (PGP) bacteria’ have already been commercialized as agricultural inoculants (Boleta et al., 2020; Wen et al., 2021), field performance is typically inconsistent, largely due to the inability of introduced inoculants to reach a quorum density on the root, but also due to ‘selfish’ genetic repression of PGP traits when conditions are unfavourable for the bacterium (Haskett et al., 2020). Key examples of this are nitrogen fixation (i.e., conversion of N_2_ ➔ NH_3_) (Bueno Batista & Dixon, 2019) and phosphate solubilization (i.e., release of available phosphorus from organic matter) (Boukhris et al., 2016; Makarewicz et al., 2006; Shen et al., 2016), where the catalytic genes are repressed under NH_3_ and phosphorus excessive conditions, respectively.

With recent advances in the synthetic biology toolkit, engineering PGP bacteria for improved function has become a prolific area of research (Haskett et al., 2020; Ke et al., 2020). Bacteria can be genetically modified to overcome negative feedback repression on nitrogen fixation (Bueno Batista et al., 2021; Haskett, Karunakaran, et al., 2022a), and refactored PGP traits can even be transferred into new, potentially more competitive bacterial chassis (Patel & Archana, 2018; Ryu et al., 2020; Shulse et al., 2019; Temme et al., 2012; Zúñiga et al., 2018). In early‐stage engineering, transgenes are commonly overexpressed to accentuate detectable phenotypes for ease of screening under laboratory conditions. Consequentially, the increased metabolic demand can impair viability and fitness of the host bacteria, driving strong selection for silencing mutations. Utilization of tuneable genetic switches to regulate transgene expression is therefore critical to stabilize gene function and preserve the native ecological characteristics of recipient bacteria (Haskett et al., 2020). For example, implementation of an acyl‐homoserine lactone (AHL)‐mediated quorum‐sensing (QS) circuit to control the biosynthesis of the phytohormone indole‐3‐acetic acid (IAA) in an autoregulated manner permitted *Cupriavidus pinatubonensis* JMP134 to enhance root growth of inoculated *Arabidopsis thaliana* (Zúñiga et al., 2018). Similarly, QS was used to regulate nitrogen fixation in *E. coli* carrying refactored nitrogenase genes derived from *Klebsiella oxytoca*, although no plant growth promotion was assessed in this case (Ryu et al., 2020).

While QS control can permit specific activation of bacterial traits when colonizing plant roots and rhizosphere where cell density is higher than the bulk soil (D'Angelo‐Picard et al., 2004; DeAngelis et al., 2008; Dessaux et al., 2011; Wang et al., 2020), utilization of plant‐derived signals to control PGP traits has the additional capacity for exclusive coupling of plant‐microbe interactions that could prevent bacterial interactions with non‐target plants (Haskett et al., 2020). Genetic biosensors for common legume root exudate signals such as sugars, polyols, organic acids and flavonoids have been characterized in *Rhizobium leguminosarum* bv. *viciae* (*Rlv*) 3841 (Pini et al., 2017), whereas bacterial signalling molecules including opines (Mansouri et al., 2002; Mondy et al., 2014; Oger et al., 1997), phloroglucinols (Abdel‐Ghany et al., 2016) and AHLs (Fray et al., 1999) have been engineered into plants to permit more stringent signalling with bacteria.

To establish exclusive signalling between plants and microbes, we previously engineered a biosynthesis pathway for the rhizopine *scyllo‐*inosamine (SI) into barley and *Medicago* (Geddes, Paramasivan, et al., 2019b). Rhizopines, including SI and 3‐*O*‐methyl SI (3‐*O*‐MSI) are inositol‐derived compounds, naturally produced by a select few bacterial species of the *Rhizobium*, *Sinorhizobium* specifically when housed within legume nodules (Murphy et al., 1995; Wexler et al., 1995). Based on genetic in vitro biochemical assays, 3‐*O*‐MSI appears to be naturally synthesized by bacteria via a two‐step process involving an ononitol dehydrogenase encoded by *mosDEF* and an amino‐transferase encoded by *mosB* which are expressed under control of the nitrogenase master regulator NifA (Bahar et al., 1998; Geddes, Paramasivan, et al., 2019b; Rossbach et al., 1994). Bacteria capable of synthesizing rhizopines typically also harbour *moc* genes (*mocRABCDEF*) required for rhizopine catabolism (Murphy et al., 1987; Wexler et al., 1995). Thus, when 3‐*O*‐MSI is exuded from the nodules, it can act as both a carbon and nitrogen source specifically for progeny cells, enhancing nodulation competitiveness (Gordon et al., 1996; Murphy et al., 1995).

In contrast to the natural mode of rhizopine biosynthesis, engineered rhizopine‐producing (*RhiP*) plants were designed to biosynthesise SI via a modified metabolic pathway involving a constitutively expressed inositol dehydrogenase *idhA* and aminotransferase *mosB* (Geddes, Paramasivan, et al., 2019b). SI is exuded from the roots into the rhizosphere, where it can be sensed by co‐engineered bacteria carrying a SI receiver plasmid (pSIR). The pSIR plasmids were designed to mimic the natural mode of rhizopine sensing in *S. meliloti* (*Sm*) L5‐30. Rhizopine is transported into the bacterial cytoplasm by the solute‐binding protein MocB interacting with the transmembrane domain components of an ATP‐binding cassette (ABC) transporter for inositol derived from *Rlv* 3841. Rhizopine binds to the transcription factor MocR, which activates expression from the rhizopine‐inducible promoter P*mocB* (Geddes, Paramasivan, et al., 2019b; Haskett, Paramasivan, et al., 2022b; Rossbach et al., 1994). Using a SI biosensor to drive expression of the nitrogenase master regulator *nifA*, we established SI‐dependent activation of nitrogen fixation by our model strain *Azorhizobium caulinodans Ac*LP colonizing *RhiP* barley roots (Haskett, Paramasivan, et al., 2022b). Moreover, we developed a genetic switch involving a unidirectional adenylyl transferase (Schnabel & Sattely, 2021) that prevents *Ac*LP from assimilating NH_3_ derived from nitrogen fixation specifically when sensing rhizopine (Haskett, Karunakaran, et al., 2022a). Although engineered *Ac*LP cells can sense SI and activate gene expression, only a subpopulation of the bacteria occupying *RhiP* barley roots activate gene expression and express nitrogenase, resulting in population‐level nitrogenase activity that equates to approximately 15% that of a wild‐type system. Thus, further optimisation of rhizopine‐signalling circuitry remains necessary to improve the functionality of this prototype ‘synthetic symbiosis’.

In this work, we perform a detailed functional analysis of rhizopine‐inducible gene expression in bacteria, identifying a new SI‐inducible promoter P*mocD* and identifying key elements in SI‐dependent promoters. We explore the host‐range of the most recently developed SI biosensor plasmid pSIR02 and demonstrate that excessive rhizopine uptake stemming from overexpression of the transmembrane components *intBC* has detrimental effects on host‐cell growth. By tuning expression of *intBC*, we constructed a new biosensor plasmid pSIR05 that has minimal impact on host cell growth in vitro and exhibits markedly improved stability of expression on the surface of *RhiP* barley roots compared to the previously described biosensor plasmid pSIR02. However, our model strain *Azorhizobium caulinodans Ac*LP carrying pSIR02 or pSIR05 colonized *RhiP* barley roots with 50% effectiveness compared to the wild‐type strain, indicating that there are still factors to be managed to reduce negative impacts on the host. Overall, this work establishes more robust plant‐dependent control of bacterial gene expression and highlights the challenges remaining to further improve rhizopine‐signalling circuitry.

## RESULTS

### Characterization of two rhizopine‐inducible promoters P*mocB* and P*mocD*

**FIGURE 1:**
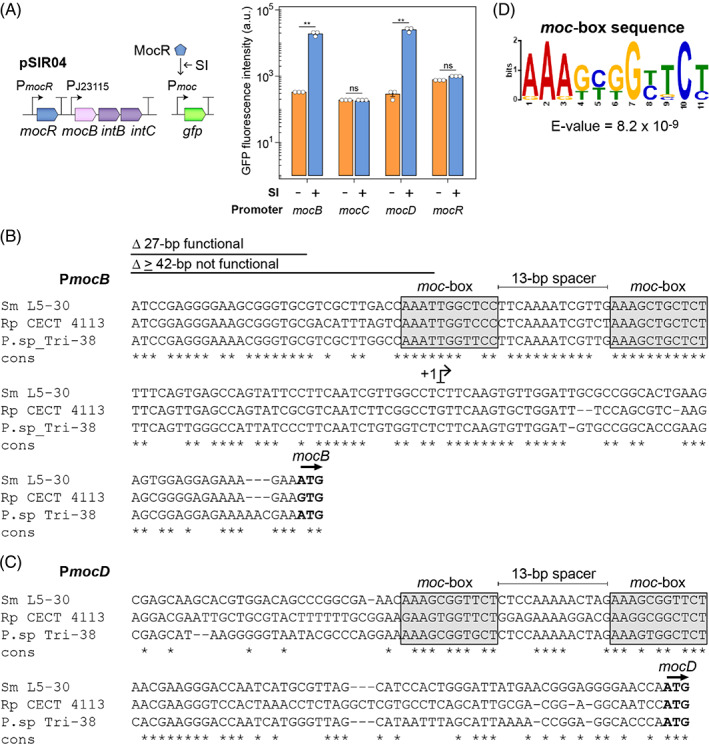
Analysis of rhizopine‐inducible promoters. (A) The rhizopine receiver plasmid pSIR04 and compatible promoter GFP fusion plasmids were each mobilized into *Azorhizobium caulinodans Ac*LP for assessment of rhizopine‐inducible expression by flow‐cytometry after 24‐h induction. Error bars represent one SEM (*n =* 3). Independent two‐tailed student's *t*‐tests were used to compare means. Not significant (ns *p* > 0.05), **p < 0.01. (B) Alignment of three unique *mocB* promoter sequences from *Sinorhizobium meliloti* (*Sm*) L5‐30, *Rhizobium pisi* (*Rp*) CECT 4113, and *phyllobacterium* (*P*) sp. Tri‐38. The transcriptional start site (+1) was identified by 5′‐RACE (see Figure S2) and a promoter reduction experiment was performed to determine the minimal functional promoter region (see Figure S3). (C) Alignment of three unique *mocD* promoter sequences from *Sm* L5‐30, *Rp* CECT 4113, and *P*. sp. Tri‐38. (D) MEME output for the identification of a loosely conserved 11‐bp direct repeat motif separated by a 13‐bp spacer in the three unique *mocB* and *mocD* promoters

In rhizopine‐catabolizing bacteria, the transcription factor MocR facilitates rhizopine‐dependent expression from the P*mocB* promoter positioned upstream of *mocBA* (Bahar et al., 1998; Geddes, Paramasivan, et al., 2019b; Rossbach et al., 1994). Considering that rhizopine catabolism additionally involves the *mocC* and *mocDEF* genes (Bahar et al., 1998; Rossbach et al., 1994), which did not appear to be transcriptionally coupled to *mocBA* (Figure S1), we predicted that one or more alternative rhizopine‐inducible promoters(s) may be present in this catabolic region. To identify these promoters, we fused the DNA regions directly upstream of *mocR*, *mocD*, *mocB* and *mocC* from *Sinorhizobium meliloti* (*Sm*) L5‐30 to a GFP reporter gene in pOGG024 and transferred the resulting plasmids into *Ac*LP carrying the compatible *scyllo*‐inosamine receiver plasmid pSIR04 (Figure 1A). As expected, GFP expression was induced from the *mocB* promoter 57.94‐fold (SE ± 1.22) by addition of 10 μM SI to the culture (Figure 1A). We also observed 93.49‐fold (SE ± 19.02) rhizopine‐induced GFP expression from the *mocD* promoter upstream of *mocDEF* but did not observe rhizopine‐inducible GFP expression from the *mocR* or *mocC* promoters, which were constitutively expressed.

To identify the minimal functional region of the *mocB* promoter, we performed 5′‐RACE on *Ac*LP carrying pSIR02 grown in the presence of 10 μM SI and identified a single transcriptional start site (TSS) located 46‐bp upstream of the *mocB* start codon (Figure 1B and Figure S2). We also performed a promoter reduction experiment where 25–27 bp increments of DNA were sequentially deleted from the 5′‐end of P*mocB* fused to a *luxCDABE* (*lux*) cassette and screened for functionality by bioluminescence assays in *Rlv* 3841 carrying the rhizopine biosensor plasmid pSIR02b in the presence or absence of 10 μM SI (Figure S3 and Figure 1B). Deletion of up to 27‐bp from the *mocR* start codon encoded divergently to *mocB* resulted in functional SI‐inducible *lux* expression; however, the deletion of 42‐bp or more completely abolished activity of the promoter, suggesting this region may harbour one or more important functional sites. Thus, we predicted that the entire functional P*mocB* promoter region is comprised of 72‐bp of DNA.

Although a MocR binding site had not yet been identified in the SI‐inducible promoters, binding sites for four structurally related MocR‐type TFs had been described, each consisting of distinct direct or inverted repeat sequences of at least 6‐bp in length (Tramonti et al., 2018). To identify potential MocR binding sites, we probed the three distinct *mocB* and *mocD* promoter regions from *Sm* L5‐30, *Phyllobacterium* sp. Tri‐38, and *Rhizobium pisi* CECT 4113 (Figure S1) for repeat motifs using MEME (Figure 1B–D). Two loosely conserved 11‐bp direct repeats of the motif 5′‐AAAGYGGYTCT‐3′ (*E* = 8.2 × 10^9^) were identified in the query promoter sequences separated by 13‐bp in each case. The motif identified at the 5′‐end of the *mocB* promoter was positioned in the region that was found to be essential for functionality in our promoter reduction assay (Figure S2 and Figure 1B), strongly suggesting that this motif comprised a MocR binding site.

### Host‐range of rhizopine biosensor plasmids

**FIGURE 2:**
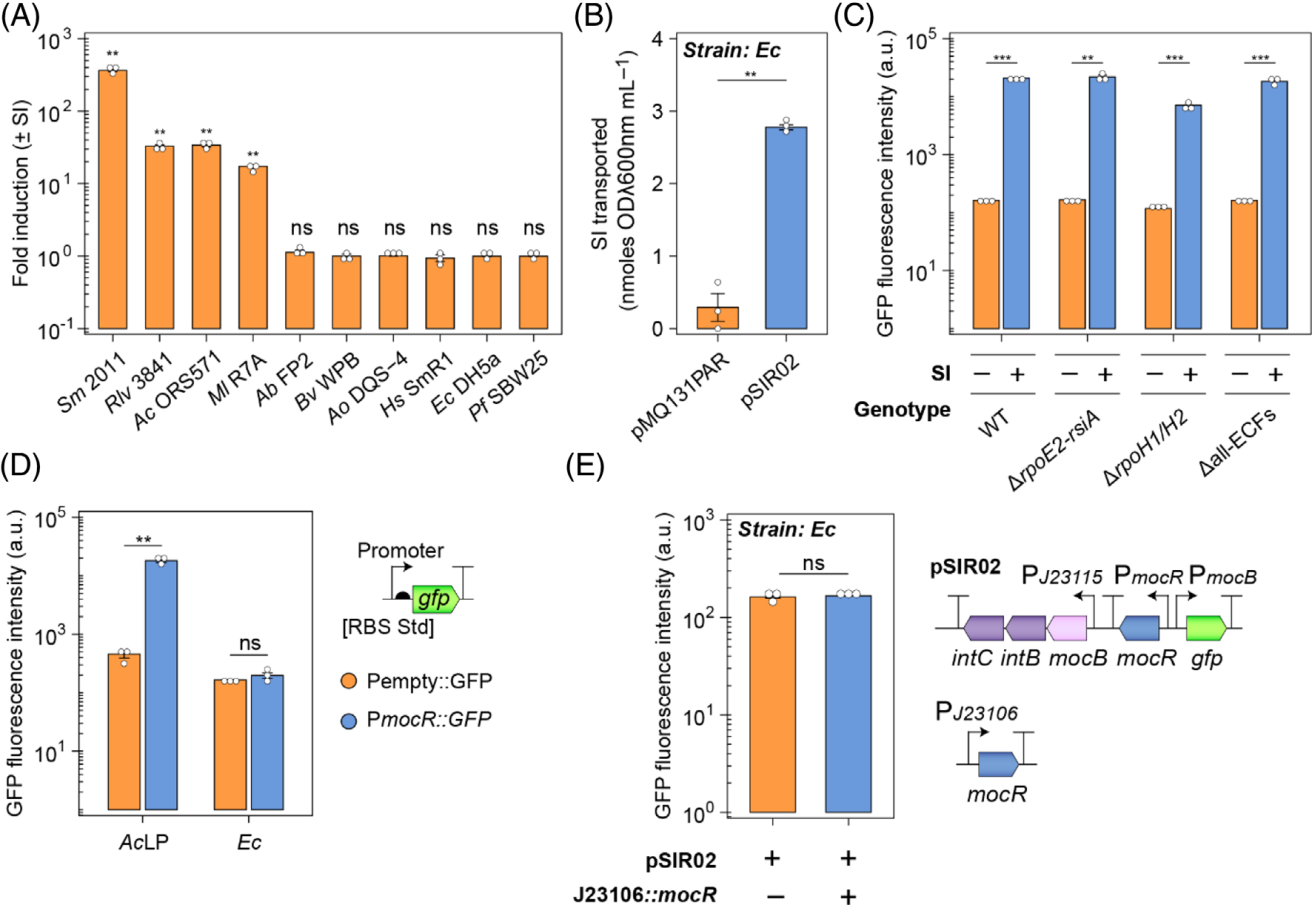
Functionality of rhizopine biosensor plasmids is restricted to rhizobial alpha‐proteobacteria. (A) SI‐inducible GFP expression was tested in a range of bacteria (*Sm*, *Sinorhizobium meliloti*; *Rlv*, *Rhizobium leguminosarum* bv. *viciae*; *Ac*, *Azorhizobium caulinodans*; *Ml*, *Mesorhizobium loti*; *Ab*, *Azospirillum brasilense*; *Bv*, *Burkholderia vietnamienses*; *Ao*, *Azoarcus olearius*; *Hs*, *Herbaspirillum seropedicae*; *Ec*, *E. coli*; *Pf*, *Pseudomonas stuzeri*) carrying the rhizopine biosensor plasmid pSIR02. Fold‐induction was calculated as relative fluorescence units (GFP fluorescence intensity/OD λ600nm) for bacteria grown with 10 μM SI supplemented into the growth media divided by RFU for non‐induced cells. (B) SI transport assay for *E. coli* DH5a carrying pSIR02. (C) Test of rhizopine‐inducible GFP expression in *Sm* CL150 and extra‐cytoplasmic sigma factor mutants carrying pSIR02. (D) Assessment *mocR* promoter functionality in *E. coli* DH5a using transcriptional reporter plasmids. Pempty is a randomly generated 30‐bp nucleotide sequence that serves as a negative control. (E) A constitutively expressed copy of *mocR* was introduced into *E. coli* DH5a carrying pSIR02 to test whether this would permit functionality of the rhizopine‐inducible GFP expression. Error bars represent one SEM (*n =* 3). Independent two‐tailed students t‐tests were used to compare means, except for in panel (a) where fold‐induction for each strain was subject to Bonferroni adjusted one sample t‐tests under the null hypothesis that μ = 1. Not significant (ns *p* > 0.05), **p < 0.01, ***p < 0.001. Bacteria in reporter assays were induced for 24‐h prior to measurement.

We had previously demonstrated that rhizopine biosensor plasmids are functional in *Ac*, *Sm* and *Rlv*. Here, we tested the functionality of pSIR02 (Figure 2A) in a more diverse range of alpha‐, beta‐ and gamma‐proteobacteria and found that SI‐inducible GFP expression was restricted to the rhizobial alpha‐proteobacteria (Figure 2A). Dysfunctionality outside of rhizobia could be explained by (1) inability to transport rhizopine; (2) incompatibility of the *mocR/mocB* promoters; or (3) incompatibility of MocR with resident transcriptional machinery. To test the first hypothesis, we used *E. coli* DH5a as a model organism and demonstrated using a previously validated SI transport assay (Haskett, Paramasivan, et al., 2022b), that SI transport was functional in this strain (Figure 2B). To explore the second and third hypotheses, we used a functional genetics approach to test whether a rhizobial sigma (σ) factor might be involved in transcriptional initiation from P*mocB*. We mobilized pSIR02 into *Sm* CL150 and deletion mutants for *rpoH1/H2* (RFF231), *rpoE2‐rsiA* (RFF164), and all 11 ECFs (RFF625c) (Lang et al., 2018) and found that SI‐inducible GFP expression was functional in each, indicating that these sigma factors were not necessary for function (Figure 2C). Although mutants for the housekeeping σ^70^ factor *rpoD* and alternative σ^70^ factor *rpoN* were not available in this strain, it seems plausible that either of these might be utilized in this case, although we were unable to identify any clear consensus elements (Dombrecht et al., 2002; MacLellan et al., 2006) for either in the *mocD* or *mocB* promoters.

We next explored whether *mocR* was expressed in *E. coli* by constructing two reporter plasmids, one where the *mocR* promoter was transcriptionally fused to GFP with a synthetic ribosome binding site (pOPS2003), and the other where a randomly generated 30‐bp of DNA was fused to GFP with the same RBS to as a negative control (pOPS1955) (Figure 2D). As expected, GFP fluorescence was higher for *Ac*LP strains carrying the P*mocR*::GFP fusion compared to the negative control, whereas for *E. coli* DH5a, there was no difference in GFP fluorescence between strains carrying either reporter plasmid, indicating that *mocR* was not expressed. We subsequently drove expression of *mocR* from a synthetic J23106 Anderson promoter (http://parts.igem.org/Promoters/Catalog/Anderson) in *E. coli* carrying pSIR02, but still did not detect SI‐inducible P*mocB*::GFP expression in the strain grown in the presence of SI (Figure 2E). Thus, we concluded dysfunctionality of the SI biosensor in diverse bacteria is due to incompatibility of the *mocR/mocB* promoters and(or) MocR with resident transcriptional machinery such as RNA polymerase or sigma factors.

### Tuned expression of rhizopine uptake genes improves biosensor functionality

**FIGURE 3:**
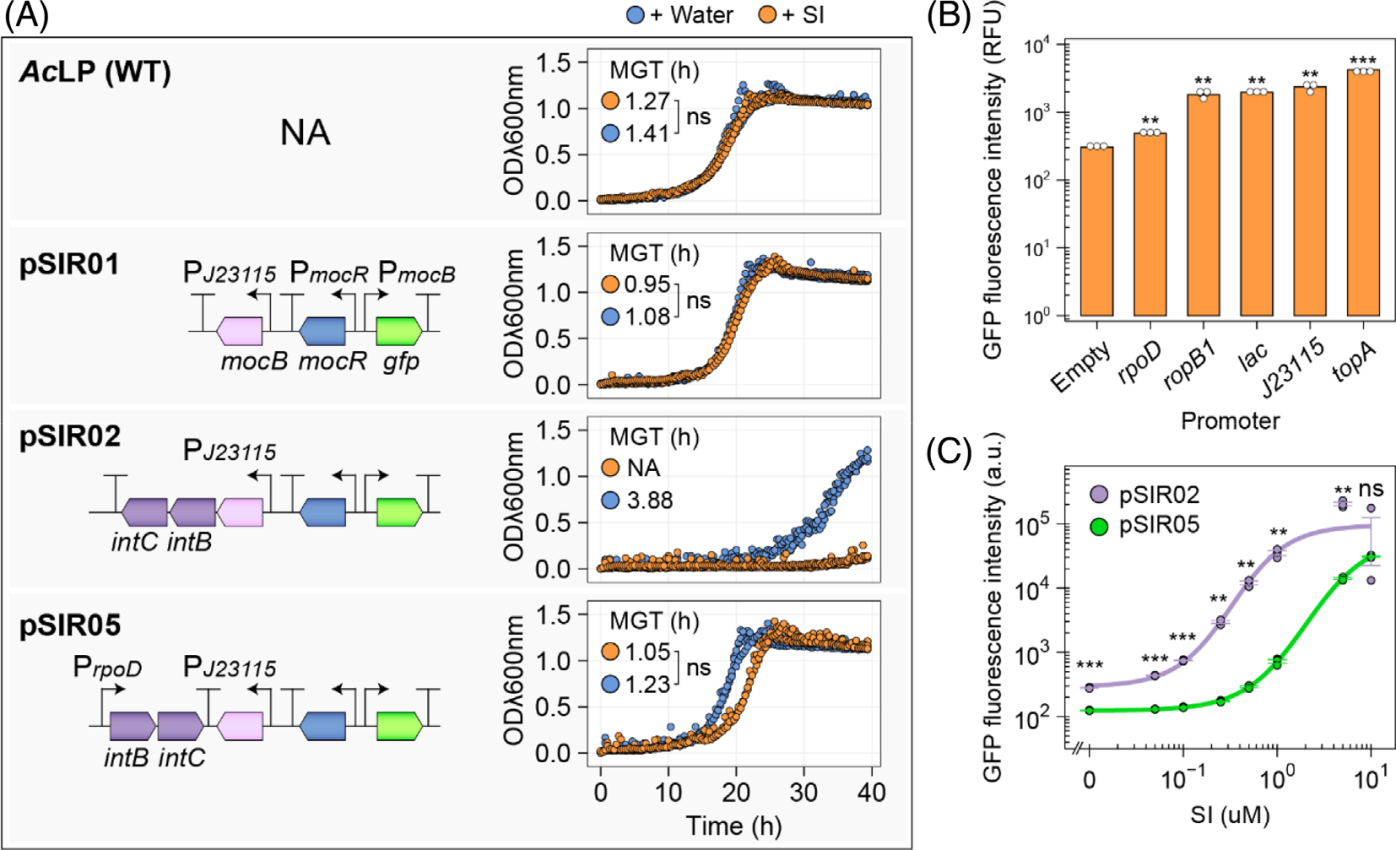
Tuning *intBC* expression for improved biosensor functionality. (A) Growth curves for *Azorhizobium caulinodans Ac*LP and strains carrying rhizopine biosensor plasmids in UMS media supplemented with 10 mM NH_3_Cl as a sole source of nitrogen and 20 mM Na succinate as a sole carbon source. Growth statistics including mean generation time (MGT) was calculated using the R package Growthcurver. (B) a series of promoters described in *Sinorhizobium meliloti* (MacLellan et al., 2006) were transcriptionally fused to GFP and tested for strength of GFP expression using fluorescence assays. RFU is defined as relative fluorescence units. Pempty is a randomly generated 30‐bp nucleotide sequence that serves as a negative control. (C) Dose response curve for GFP induction in *Ac*LP carrying pSIR02 or pSIR05. Bacteria were induced for 24‐h prior to measurement. Error bars represent one SEM (*n =* 3). Independent two‐tailed students t‐tests were used to compare means with Pempty serving as a reference group for comparisons in panel (B). Not significant (ns *p* > 0.05), **p < 0.01, ***p < 0.001

We previously combined the *Sm* L5‐30 solute‐binding protein MocB together transmembrane components of the *Rlv*3841 ABC inositol transporter IntBC to form a hybrid ABC rhizopine uptake system (Haskett, Paramasivan, et al., 2022b). Because constitutively active transport of carbon substrates can result in overaccumulation and subsequently toxicity, we analysed growth in free‐living culture of WT *Ac*LP and strains carrying rhizopine biosensor plasmids harbouring only *mocB* (pSIR01) which do not transport SI, or *mocB‐intBC* (pSIR02) expressed from the weak synthetic J23115 Anderson promoter which transport SI (Haskett, Paramasivan, et al., 2022b). The mean generation time (MGT) and carrying capacity (k, corresponding to the maximum ODλ600nm) of *Ac*LP carrying pSIR01 cells grown in the presence or absence of 10 μM SI were comparable to wild‐type *Ac*LP, whereas both the MGT and k of *Ac*LP cells carrying pSIR02 was defective and this was further accentuated by addition of 10 μM SI to the growth media, presumable due to excessive accumulation of SI (Figure 3A and Data S1).

Our SI biosensor plasmids pSIR01‐02 carry multi‐copy pBBR1 replicons. Thus, we theorized that reducing the copy number of rhizopine transport genes could reduce the rate of SI transport to alleviate toxicity, while retaining the capacity for SI perception. We tested this by transferring the rhizopine transport (*intBC* and *mocB*) and perception loci (*mocR*, P*mocB::GFP*) from pSIR02 onto a mini‐Tn7 plasmid (pOPS1740) and integrating this in single copy into the chromosome of *Ac*LP. The resulting strain *Ac*SIR exhibited a MGT and k comparable to the wild‐type control *Ac*LP in the presence and absence of 10 μM SI; however, we did not detect SI‐inducible GFP expression (Figure S4 and Data S2). To confirm that SI transport was functional in *Ac*SIR, we introduced pSIR01 into *Ac*SIR and found that while the resulting strain retained wild‐type growth characteristics, we were able to detect SI‐induced GFP expression (Figure S4 and Data S2), suggesting that reduced *intBC* expression was alone sufficient alleviate SI toxicity. Furthermore, this result suggested that the failure of *Ac*SIR to show SI‐inducible GFP expression was due to insufficient levels of the MocR or MocB protein(s) and potentially dosage of the cognate P*mocB*::GFP cassette. Thus, we opted to identify promoters weaker than J23115 that could be used to drive *intBC* expression on a multi‐copy pBBR1 replicon SI biosensor.

We selected a range of promoters previously characterized in *Sm* (MacLellan et al., 2006) and made a series of transcriptional GFP fusions to test expression levels in *Ac*LP (Figure 3B). P*rpoD* was identified as the weakest strength promoter tested, exhibiting GFP fluorescence 4.84‐fold (SE ± 0.02) less than the synthetic Anderson promoter J23115 previously used to drive *mocB‐intBC* expression on pSIR02 and 1.60‐fold (SE ± 0.04) higher than the negative control. Thus, we utilized the P*rpoD* to drive *intBC* expression on a reconstructed SI‐biosensor plasmid pSIR05 that was otherwise identical to pSIR02 (Figure 3A). When pSIR05 was mobilized into *Ac*LP, the resulting strain exhibited wild‐type growth characteristics when grown in the presence or absence of SI (Figure 3A) and importantly, the strain retained the ability to perceive SI and activate GFP expression from P*mocB* (Figure 3C), although perception was less sensitive than that of pSIR02, presumably due to a reduced rate of SI transport. GFP expression from P*mocB* was induced 248‐fold (SE ± 3.49) for pSIR05 at 10 μM SI which was not significantly different from that of pSIR02 (*p =* 0.959).

### In situ SI‐dependent expression on *RhiP* barley

**FIGURE 4:**
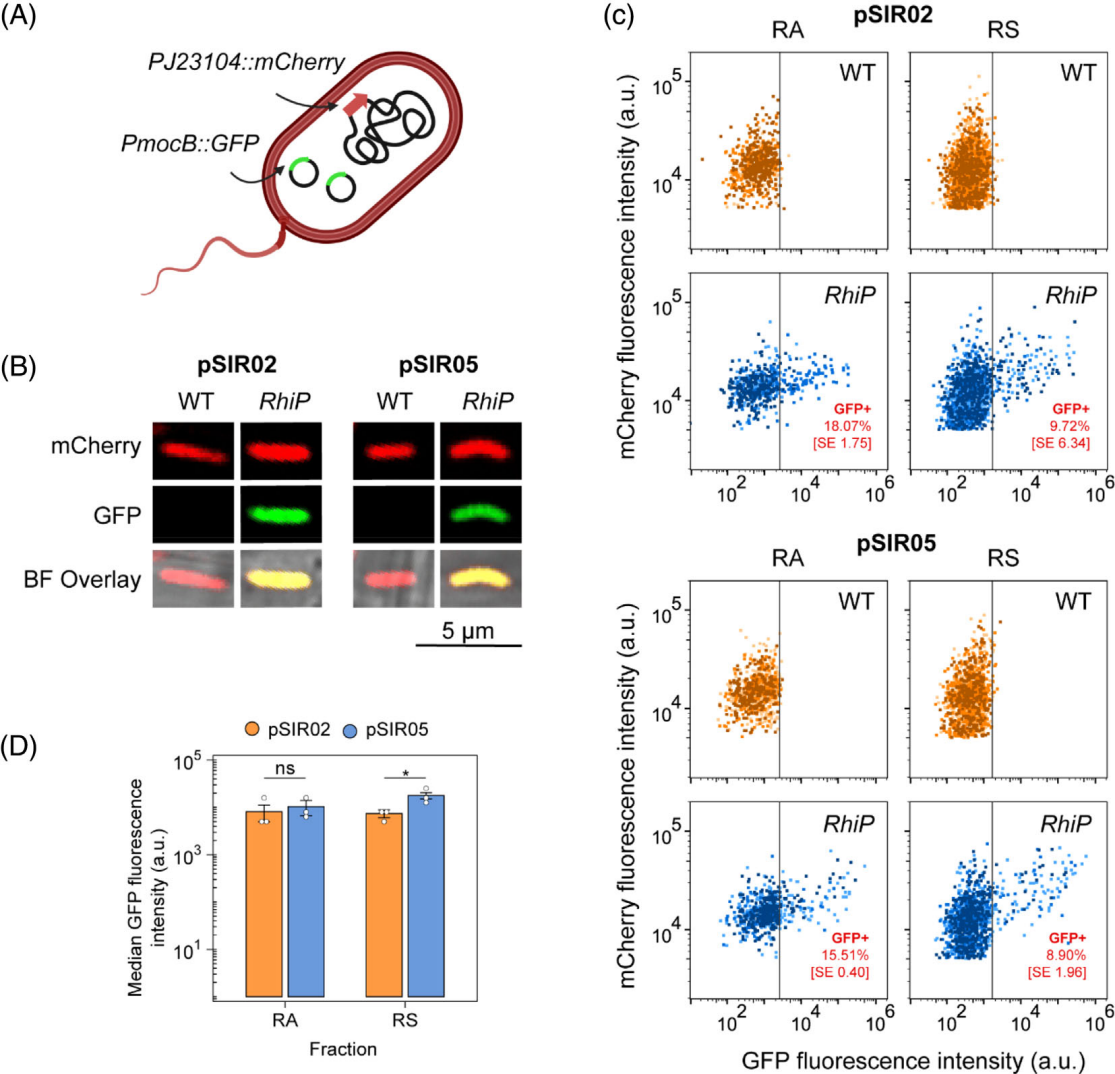
In situ rhizopine‐dependent expression on *RhiP* barley roots. (A) Diagram of *Ac*Cherry, a derivative of *Azorhizobium caulinodans Ac*LP harbouring a constitutively expressed mCherry gene integrated in single‐copy into the chromosome. (B) Confocal microscopy images of single *Ac*Cherry cells carrying the rhizopine biosensor plasmids pSIR02 or pSIR05 colonizing the root surface of *RhiP* barley. Approximately 2 × 10^8^ bacteria were inoculated onto *RhiP* barley, which was grown 7‐dpi prior to harvest. (C) Flow‐cytometry quantification of rhizopine‐inducible GFP expression in populations of *Ac*Cherry cells carrying pSIR02 or pSIR05 colonizing the root associated (RA, defined as root surface and endosphere) and rhizosphere (RS, defined as sand surrounding the roots) fractions of wild‐type (WT) and *RhiP* barley roots (*n =* 3, shades of orange represent each replicate). Only bacteria exhibiting mCherry fluorescence >5000 a.u. (mCherry+) were considered for analysis. The upper 99th percentile of GFP fluorescence intensity in mCherry+ bacteria isolated from wild‐type barley was used to define a threshold for GFP+ cells. (D) Median GFP fluorescence intensity from the GFP+ populations of *Ac*Cherry carrying pSIR02 or pSIR05. Error bars represent one SEM. Independent two‐tailed student's *t*‐tests were used to compare means. Not significant (ns *p* > 0.05), *p < 0.05

Our SI biosensors were ultimately intended for in situ control of bacterial gene expression on *RhiP* barley roots. Therefore, we sought to compare functionality of our newly constructed SI biosensor plasmid pSIR05 with pSIR02 in this condition. Both plasmids were first mobilized into *Ac*Cherry, a derivative of *Ac*LP carrying a mini‐Tn7 integrated mCherry gene expressed from the constitutive Anderson promoter PJ23104 which permits the identification of bacteria colonizing roots of barley plants (Figure 4A,B). Wild‐type (WT) and *RhiP* barley plants were inoculated with approximately 2 × 10^8^ cells and grown in fire sand for 7‐days post inoculation (dpi). SI‐inducible P*mocB*::GFP expression was assessed in single cells by flow‐cytometry for bacteria exhibiting mCherry fluorescence >5000 a.u. in two fractions of WT and *RhiP* barley roots; RA root‐associated (occupying the root surface and endosphere) and RS rhizosphere (occupying the sand surrounding the root) (Haskett et al., 2021; Haskett, Paramasivan, et al., 2022b). The upper 99th percentile of GFP fluorescence intensity for bacterial populations occupying wild‐type barley plants was used to establish a threshold value for ‘GFP+’ cells isolated from *RhiP* barley plants and based on this threshold, we determined that 15.51% (SE ± 0.40) and 8.90% (SE ± 1.96) of *Ac*Cherry cells carrying pSIR05 were GFP+ in the RA and RS fractions, respectively, which was not significantly different from *Ac*Cherry cells carrying pSIR02 in either fraction (RA *p =* 0.856, RS *p =* 0.784) (Figure 4C). There was also no difference in the median GFP fluorescence intensity measured for *Ac*Cherry cells carrying pSIR05 compared to pSIR02 in the RA fraction, whereas in the RS fraction, median GFP fluorescence intensity was higher for pSIR05 compared to pSIR02 (Figure 4D), indicating in situ SI‐inducible expression was stronger for cells carrying pSIR05 in this fraction.

### Colonization effectiveness and stability of expression on *RhiP* barley

We next performed colonization assays on WT *Ac*Cherry and cells carrying pSIR05 or pSIR02 by inoculating 10^5^ cells of each strain individually onto *RhiP* barley and growing the plants for 7‐dpi prior to counting bacteria with mCherry fluorescence >5000 a.u. by flow‐cytometry. We found that the *Ac*Cherry bacteria carrying pSIR05 or pSIR02 colonized both RA and RS fractions with equal effectiveness. While the total number of cells carrying pSIR05 (*p* = 0.33) or pSIR02 (*p* = 0.56) isolated from the RA fraction of *RhiP* barley plants was no different than the wild‐type, the total number of cells carrying pSIR05 (*p* = 0.01) and pSIR02 (*p* < 0.01) isolated from the RS fraction was approximately 50% that of the wild‐type bacteria, suggesting that carrying an SI biosensor plasmid may convey a root surface and endosphere colonization defect.

**FIGURE 5:**
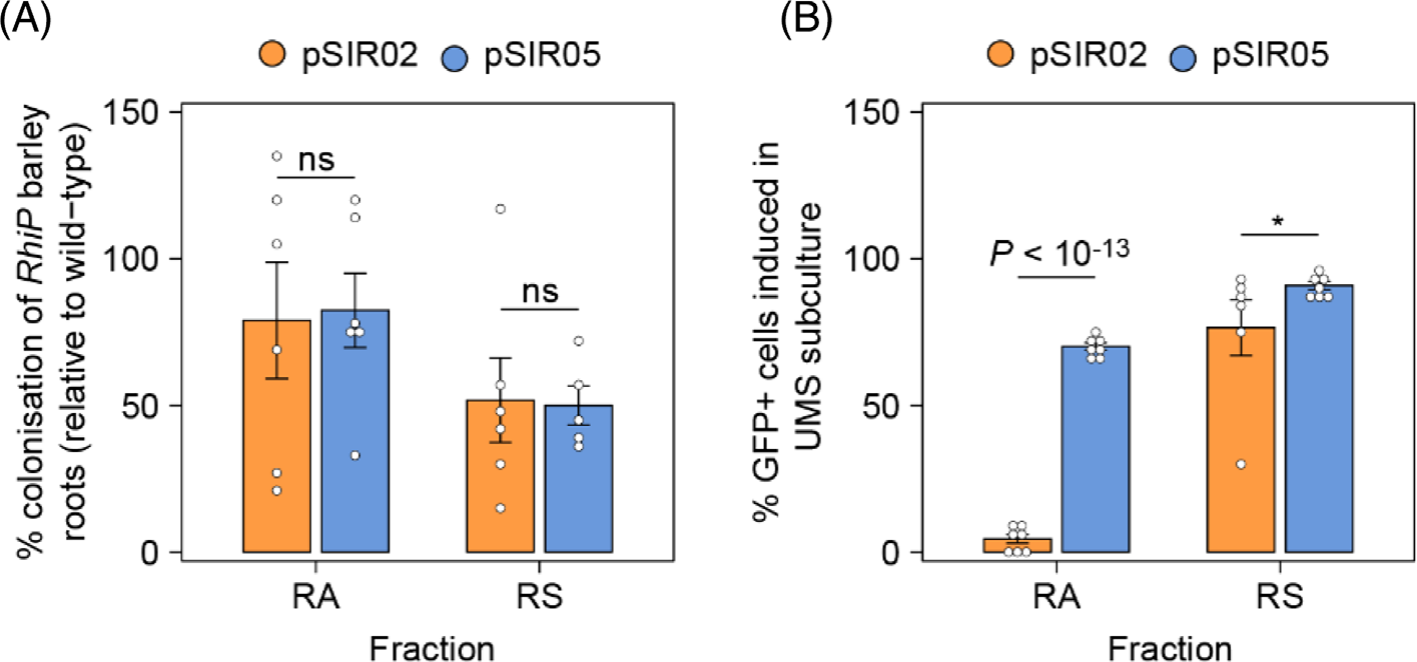
Colonization effectiveness and stability of expression on *RhiP* barley roots. (A) Approximately 10^5^ bacteria for each strain were independently inoculated onto *RhiP* barley, which was grown 7‐dpi prior to harvest. Bacteria isolated from the root associated (RA, defined as root surface and endosphere) and rhizosphere (RS, defined as sand surrounding the roots) fractions exhibiting mCherry fluorescence >5000 a.u. (mCherry+) were counted by flow‐cytometry. Colonization effectiveness was measured as the percentage of *Ac*Cherry cells carrying a biosensor plasmid compared to the wild‐type control, *Ac*Cherry devoid of plasmids. (B) Analysis of silencing analysis for *Ac*Cherry carrying pSIR02 and pSIR05. Cell suspensions recovered from the colonization assays were seeded 1:10 in UMS media and induced for 24‐h with 10 μm SI prior to analysis by flow‐cytometry. The upper 99th percentile of GFP fluorescence intensity in a non‐induced free‐living culture (Figure 3C) was used to define a threshold for GFP+ cells. Error bars represent one SEM. Independent two‐tailed student's *t*‐tests were used to compare means (*n =* 7). Not significant (ns *p* > 0.05), *p < 0.05

To assess the stability of SI‐inducible expression in *Ac*Cherry cells carrying pSIR05 and pSIR02, we sub‐cultured cells re‐isolated from each fraction of our colonization assays into UMS media and induced them for 48‐h with 10 μM SI. SI‐induced P*mocB*::GFP expression was assessed by flow‐cytometry in bacteria exhibiting mCherry fluorescence >5000 a.u. after defining a GFP+ threshold as the upper 99th percentile of GFP fluorescence intensity in a non‐induced free‐living culture (Figure 3C). We observed very little loss of capacity for P*mocB*::GFP expression in *Ac*Cherry cells carrying pSIR05 or pSIR02 recovered from the RS fraction; however, while 70% of *Ac*Cherry cells carrying pSIR05 isolated from the RA fraction retained the capacity for SI‐inducible P*mocB*::GFP expression, only 3% *Ac*Cherry cells carrying pSIR02 from the RA fraction retained this capacity (Figure 5B). This loss of functionality may be due to silencing or plasmid loss, although we expect the former scenario is most likely considering that the pSIR plasmids carry PAR genes for plasmid maintenance. Overall, we conclude that the newly constructed SI biosensor plasmid pSIR05 conveys more stable perception of SI on the root surface and in the endosphere compared to pSIR02.

## DISCUSSION

Genes required for rhizopine catabolism in soil bacteria are organized into four transcriptional units, *mocDEF*, *mocR*, *mocBA* and *mocC* (Bahar et al., 1998; Rossbach et al., 1994) (Figure S1), with the *mocB* promoter being rhizopine‐inducible (Geddes, Paramasivan, et al., 2019b) (Figure 1A). Here we identified that the *mocD* promoter was additionally rhizopine‐inducible (Figure 1A), expanding our toolkit for engineering rhizopine‐dependent control and allowing us to probe the sequence data for functional components of rhizopine‐inducible expression systems (Figure 1B–D). Both the *mocB* and *mocD* promoters harboured two loosely conserved 11‐bp motifs 5′‐AAAGYGGYTCT‐3′ herein termed ‘*moc*‐boxes’ that are separated by 13‐bp of DNA and we propose act as a binding sites for MocR. The arrangement of the repeat motifs is consistent with those identified for other MocR‐like transcription factors (Tramonti et al., 2018), such as GabR in *Bacillus subtlis* (Belitsky, 2004; Belitsky & Sonenshein, 2002), *PdxR* in *Corynebacterium glutamicum* (Jochmann et al., 2011) and TauR in *Rhodobacter capsulatus* (Wiethaus et al., 2008). We also found that *mocR* and *mocC* were constitutively expressed. While this is perhaps not surprising for a transcription factor such as *mocR*, constitutive expression is atypical of a gene such as *mocC*, which encodes a protein of unknown function that is essential for catabolism of both SI and 3‐*O*‐MSI (Bahar et al., 1998; Rossbach et al., 1994). This mode of regulation may be attributable to a potential role for mocC in the metabolism of alternative carbon sources, such as *myo‐*inositol, as was previously postulated (Bahar et al., 1998; Fry et al., 2001; Rossbach et al., 1994).

Utilizing rhizopine signalling to establish plant host‐dependent control of gene expression in diverse bacteria is highly desirable. However, we demonstrated here that functionality of rhizopine biosensor plasmids is restricted to rhizobial alpha‐proteobacteria (Figure 2A). This restricted function was not likely due to an inability of distantly related bacteria to transport rhizopine, as we demonstrated that SI transport was functional in *E. coli* carrying pSIR02. Moreover, restricted functionality was not due to a requirement for accessory sigma factors present in *Sinorhizobium* (Lang et al., 2018) (Figure 2C) that might be absent in more diverse bacteria. Instead, it seems likely that the lack function of rhizopine biosensors outside of the rhizobial alpha‐proteobacteria might reflect incompatibility of transcriptional machinery and promoters. We have observed the same effect when transferring various regulated rhizobial promoters into *E. coli*, and this has also been documented elsewhere (Bae & Stauffer, 1991). Thus, expanding the host‐range of rhizopine signalling beyond the alpha‐proteobacteria may require more complex development of a hybrid RNA‐polymerase (Peck et al., 2002), an aptamer (Ruscito & DeRosa, 2016; Spöring et al., 2020) or an engineered metabolite‐responsive transcription factor (Wan et al., 2019; Younger et al., 2018).

We previously demonstrated that MocB together with the transmembrane components of the *Rlv*3841 ABC inositol transporter IntBC, form a hybrid ABC rhizopine uptake system that is essential for sensitive SI‐inducible gene expression in *Ac*LP (Haskett, Paramasivan, et al., 2022b). Here, we found that overexpression of the transmembrane components *intBC* in *Ac*LP results in defective growth in the absence of SI, and strongly inhibits growth when SI was added to the growth media (Figure 3A). However, this rhizopine toxicity was alleviated by reducing copy number or expression level of *intBC*. Reduced expression of *intBC* using the newly constructed SI biosensor pSIR05 yielded a *Ac*LP strain that perceived SI with slightly less sensitivity compared to *Ac*LP carrying pSIR02 (Figure 3B), presumably due to a reduced rate of SI transport. As *Ac*LP does not carry SI catabolic genes (Murphy et al., 1987; Rossbach et al., 1994) and does not degrade SI, it seems plausible that the strain would exhibit a more sensitive response to SI when given a longer incubation period to accumulate the inducer. This notion is supported by the finding that SI‐inducible GFP expression was equally as effective for *Ac*Cherry carrying pSIR02 or pSIR05 when inoculated at high cell density onto *RhiP* barley roots (Figure 4) where there is very little SI present (Haskett, Paramasivan, et al., 2022b).

While performance of the pSIR02 and pSIR05 rhizopine biosensors was similar when the host bacteria were inoculated onto *RhiP* barley at high cell density (i.e., 2 × 10^8^ cells), pSIR05 exhibited markedly improved stability of expression on the root surface and endosphere fraction when the host cells were inoculated at low cell density (i.e., 10^5^ cells) and were required to proliferate. This results in likely reflective of the reduced expression of *intBC* on pSIR05, which alleviates overaccumulation of SI. We have already demonstrated that T1 *RhiP* plants that produce approximately sevenfold more SI than the T2 plants used in this work, can elicit a threefold increase in the proportion of bacteria perceiving rhizopine (Haskett, Paramasivan, et al., 2022b). Thus, with stable rhizopine‐inducible expression in the bacteria, it is now feasible to attempt further elevating of rhizopine production by *RhiP* plants to increase the percentage of bacterial cells occupying roots that perceive rhizopine and activate gene expression. Achieving this will be a key factor in establishing more robust plant‐dependent control of engineered bacterial traits such as nitrogen fixation and ammonia excretion (Haskett, Karunakaran, et al., 2022a). In addition, we have observed here that bacteria carrying rhizopine biosensor plasmids are mildly defective in colonization of *RhiP* barley, and thus alleviating this colonization defect will be critical to establishing more robust control of engineered traits in future work.

## EXPERIMENTAL PROCEDURES

### Bacterial strains, plasmids and molecular techniques

Bacteria used in this study (Table S1) were cultured in TY (Beringer, 1974) or UMS (Brown & Dilworth, 1975; Haskett, Paramasivan, et al., 2022b; Poole et al., 1994). Plasmids (Table S2 and Data S2) were constructed using HiFi assembly (New England Biolabs) or BEVA modular golden‐gate assembly (Geddes, Mendoza‐Suárez, & Poole, 2019a; Weber et al., 2011) and were mobilized into strains of interest via di‐parental mating with *E. coli* ST18 (Thoma & Schobert, 2009). For mini‐Tn*7* integration into the chromosome of *Ac*LP, tri‐parental matings were required to additionally mobilize the transposase helper plasmid pTNS3 (Choi & Schweizer, 2006). 5′‐RACE was performed using a 2nd Generation Roche 5′/3′ RACE Kit as per the manufacturer's recommendations.

### Growth curves

Growth curves were performed in triplicate by streaking single colonies onto 10 ml TY agar slopes and incubating for 3 days prior a single wash with PBS and harvesting by centrifugation. Washed cells were inoculated at ODλ600nm 0.01 into 500 μl UMS media in clear‐bottomed 24‐well plates. OD600λnm was monitored at 20 or 30 min intervals using an Omega FLUOstar plate reader set to shake cultures at 700 rpm at 37°C. Growth statistics were calculated using the R package GrowthCurver (Sprouffske & Wagner, 2016).

### Reporter assays in free‐living culture

Single colonies streaked in triplicate onto 10 ml TY agar slopes in 28 ml universal vials and incubated for 2–3 days. Cells were washed from slopes in PBS, pelleted by centrifugation and inoculated at OD600λnm 0.1 in 96‐deep‐well culture plates, each well containing 500 μl UMS supplemented and an inducer where appropriate. Cultures were subsequently incubated for 24‐h with rigorous shaking prior to quantification of GFP using an Omega FLUOstar (485 nm excitation and 520 nm emission, gain 1250) or bioluminescence using a Promega GloMax® multi‐detection system. Values are presented as relative fluorescence (RFU) or luminescence units (RLU), defined as GFP intensity or luminescence (counts per second)/OD600λnm. Alternatively, cell suspensions were diluted 1:100 in PBS and GFP and mCherry fluorescence was measured in single cells using an Amnis® Cellstream® flow‐cytometer equipped with autosampler. At least 20,000 events were counted for each sample and gating of bacterial populations was performed using FloJo as previously described (Haskett, Karunakaran, et al., 2022a; Haskett, Paramasivan, et al., 2022b). Median fluorescence in the population was reported in arbitrary units (a.u.).

### Rhizopine transport assay

*E. coli* cells were prepared as described for reporter assays and ODλ600nm 0.3 LB cultures were supplemented with 100 μM SI. Cultures were incubated at 37°C for 3‐h and harvested by centrifugation. The cell pellet was washed three times with 10 ml PBS to remove residual SI and finally resuspended in 500 μl PBS prior to mechanical lysis with a MP Biomedicals FastPrep instrument. Aliquots of 50 μl of the lysed cell contents were bio‐assayed for SI using *Rlv* 3841 carrying pOPS0046 and pOPS0365 as a bioluminescent sensor. SI concentrations were determined by comparison against a SI standard curve as previously described (Haskett, Paramasivan, et al., 2022b).

### Confocal microscopy

Wild‐type and T2 *RhiP* barley plants for confocal microscopy were propagated in fire sand as previously described (Haskett et al., 2021; Haskett, Paramasivan, et al., 2022b) with 10 mM KNO_3_ added to the nutrient solution. Inoculant bacteria were prepared as described for reporter assays and 2 ml of a washed ODλ600nm 0.1 cell suspension was used added to the barley growth chambers upon sewing. Plants were grown 7‐dpi prior to harvesting. Dual channel confocal images were taken of whole lateral roots of barley using a ZEISS LSM 880 Axio Imager 2 with a C‐Apochromat 40×/1.2 W Korr FCS M27 objective as previously described (Haskett et al., 2021). Excitation of GFP and mCherry was achieved using 488 (3% power) and 561 (4% power) nm lasers, respectively, and fluorescence emissions were collected using photomultiplier tube (PMT) detectors for GFP (493–598 nm, gain 580) and mCherry (detection 598–735, gain 700).

### Analysis of in situ GFP expression on barley roots

Barley plants were propagated, inoculated and grown 7‐dpi as described for confocal microscopy. *Ac*Cherry cells were isolated from barley roots and analysed as previously described (Haskett et al., 2021; Haskett, Paramasivan, et al., 2022b). Bacteria occupying the root‐associated (RA) fraction of barley roots were isolated by excising the root below the seed and vortexing the root for 1 min in 20 ml PBS to remove loosely attached bacteria. Root fresh weight was recorded prior to crushing the roots with a sterile mortar and pestle, and the crushed root was resuspended in 5 ml of PBS. The rhizosphere (RS) fraction was isolated by rinsing bacteria from the remaining sand with 20 ml of PBS and vortexing for 1 min. All samples were passed through a 40‐μm FLOMI cell strainer and RS samples were diluted 1:10 prior to analysis by flow‐cytometry. At least 200,000 events were counted for flow‐cytometry and gating of bacterial populations was performed using FloJo as previously described (Haskett, Karunakaran, et al., 2022a; Haskett, Paramasivan, et al., 2022b). Only cells exhibiting mCherry fluorescence >5000 a.u. were used for analysis and the upper 99th percentile of GFP fluorescence intensity for bacterial populations occupying wild‐type barley plants was used to establish a threshold value for ‘GFP+’ cells.

### Root colonization and in situ silencing assays

Barley plants were propagated and grown 7‐dpi as described for confocal microscopy; however, only 1 ml of an ODλ600nm 10^−4^ cell suspension was used to inoculate the plants at the time of sewing. *Ac*Cherry cells were isolated from the roots as described for analysis of in situ GFP expression on barley roots and analysed by flow‐cytometry. Cells exhibiting mCherry fluorescence >5000 a.u. were counted and the total count of mCherry+ bacteria per gram of root was back calculated for the RA and RS samples. Colonization effectiveness was then measured as a percentage, relative to wild‐type *Ac*Cherry colonizing *RhiP* barley.

To assess silencing in the isolated RA and RS populations, each was seeded 1:10 into UMS media supplemented with 10 μM SI and 100 μg ml^−1^ kanamycin to prevent contamination, then grown with rigorous shaking in a deep 96‐well plate for 48‐h. Cultures were subsequently diluted 1:100 in PBS and analysed by flow‐cytometry as described for analysis of in situ GFP expression on barley roots. In this case, the upper 99th percentile of GFP fluorescence intensity for three free‐living populations of *Ac*LP carrying pSIR02 or pSIR05 induced with 10 μM SI was used to establish a threshold value for ‘GFP+’ cells.

### Statistical data analysis

Statistical analyses were carried out using the R package Rstatix (R Core Team, 2021). Dose response curves were fitted using the R package drc (Ritz et al., 2016). Bacterial growth statistics were analysed using the R package GrowthCurver (Sprouffske & Wagner, 2016). Details of each statistical analysis are outlined in the figure captions.

## AUTHOR CONTRIBUTIONS

**Timothy Haskett:** Conceptualization (lead); data curation (lead); formal analysis (lead); funding acquisition (equal); investigation (lead); methodology (lead); project administration (equal); supervision (equal); validation (lead); visualization (lead); writing – original draft (lead); writing – review and editing (lead). **Barney Geddes:** Conceptualization (equal); formal analysis (equal); investigation (equal); methodology (equal). **Ponraj Paramasivan:** Conceptualization (supporting); methodology (supporting); resources (equal). **Patrick Green:** Investigation (supporting); methodology (supporting). **Samir Chitnavis:** Investigation (supporting); methodology (supporting). **Marta Mendes:** Investigation (supporting); methodology (supporting). **Beatriz Jorrín:** Investigation (supporting); methodology (supporting). **Hayley Knights:** Investigation (supporting); methodology (supporting). **Tahlia R. Bastholm:** Investigation (supporting); methodology (supporting). **Joshua Ramsay:** Funding acquisition (supporting); investigation (supporting); methodology (supporting); writing – original draft (supporting); writing – review and editing (supporting). **Giles Oldroyd:** Funding acquisition (equal); resources (equal); supervision (equal). **Philip Poole:** Conceptualization (equal); funding acquisition (lead); investigation (equal); project administration (equal); resources (lead); supervision (equal); writing – original draft (supporting); writing – review and editing (supporting).

## CONFLICT OF INTEREST

The authors disclose no conflicts of interest relating to this work.

## Supporting information


**Figure S1.** Synteny alignment of bacteria carrying rhizopine catabolic genes. All bacteria carrying the full suite of rhizopine catabolic genes were identified based on BLASTP analysis. A synteny alignment of the genetic regions was constructed using EasyFig. Species and genus names are as follows; *Sm Sinorhizobium meliloti; P Phyllobacterium; R. Rhizobium; Rp Rhizobium pisi; Rl Rhizobium leguminiosarum; Rlv Rhizobium leguminosarum* bv. *viciae*.
**Figure S2.** Rapid amplification of cDNA ends (5′‐RACE) of the P*mocB* promoter. The chromatogram trace aligned to the *mocB* promoter sequence indicates the transcriptional start site is a cytosine nucleotide positioned 46‐bp upstream of the *mocB* start codon.
**Figure S3.** Identification of the minimal functional P*mocB* promoter region. A series of P*mocB* promoter regions was amplified with increments of DNA removed from the 5′‐end then transcriptionally fused to a *luxCDABE* cassette in the plasmid backbone pOGG093. The plasmid was mobilized into *Rlv* 3841 carrying pSIR02b and bioluminescence was monitored in UMS cultures supplemented with 10 μM SI.
**Figure S4.** Growth curves for *Azorhizobium caulinodans Ac*LP and strains carrying a single‐copy mini‐Tn7 integrated rhizopine biosensor derived from pSIR02. Strains were grown in UMS media supplemented with 10 mM NH_3_Cl as a sole source of nitrogen and 20 mM Na succinate as a sole carbon source. Growth statistics including mean generation time (MGT) was calculated using the R package Growthcurver. (b) GFP fluorescence intensity was assessed by flow‐cytometry in UMS cultures ±10 μM SI. Error bars represent one SEM (*n =* 3). Independent two‐tailed students t‐tests were used to compare means. Not significant (ns *p* > 0.05).

**Table S1.** Bacterial strains used in this study.

**Table S2.** Plasmids used in this study.

**Data S1.** Growth statistics of strains.

**Data S2.** Plasmid construction, oligonucleotides and gene blocks.

## Data Availability

The data that support the findings of this study are available from the corresponding author upon reasonable request.
