# Supplementary material for: Rhizopine biosensors for plant‐dependent control of bacterial gene expression
Source: Environ Microbiol. 2022 Dec 4;25(2):383–96. doi: 10.1111/1462-2920.16288 (PMC10107442; doi:10.1111/1462-2920.16288)
Supplement: Supplementary file 1 — Figure S1. Synteny alignment of bacteria carrying rhizopine catabolic genes. All bacteria carrying the full suite of rhizopine catabolic genes were identified based on BLASTP analysis. A synteny alignment of the genetic regions was constructed using EasyFig. Species and genus names are as follows; Sm Sinorhizobium meliloti; P Phyllobacterium; R. Rhizobium; Rp Rhizobium pisi; Rl Rhizobium leguminiosarum; Rlv Rhizobium leguminosarum bv. viciae. Figure S2. Rapid amplification of cDNA ends (5′‐RACE) of the PmocB promoter. The chromatogram trace aligned to the mocB promoter sequence indicates the transcriptional start site is a cytosine nucleotide positioned 46‐bp upstream of the mocB start codon. Figure S3. Identification of the minimal functional PmocB promoter region. A series of PmocB promoter regions was amplified with increments of DNA removed from the 5′‐end then transcriptionally fused to a luxCDABE cassette in the plasmid backbone pOGG093. The plasmid was mobilized into Rlv 3841 carrying pSIR02b and bioluminescence was monitored in UMS cultures supplemented with 10 μM SI. Figure S4. Growth curves for Azorhizobium caulinodans AcLP and strains carrying a single‐copy mini‐Tn7 integrated rhizopine biosensor derived from pSIR02. Strains were grown in UMS media supplemented with 10 mM NH3Cl as a sole source of nitrogen and 20 mM Na succinate as a sole carbon source. Growth statistics including mean generation time (MGT) was calculated using the R package Growthcurver. (b) GFP fluorescence intensity was assessed by flow‐cytometry in UMS cultures ±10 μM SI. Error bars represent one SEM (n = 3). Independent two‐tailed students t‐tests were used to compare means. Not significant (ns p > 0.05). [file EMI-25-383-s003.docx]

**
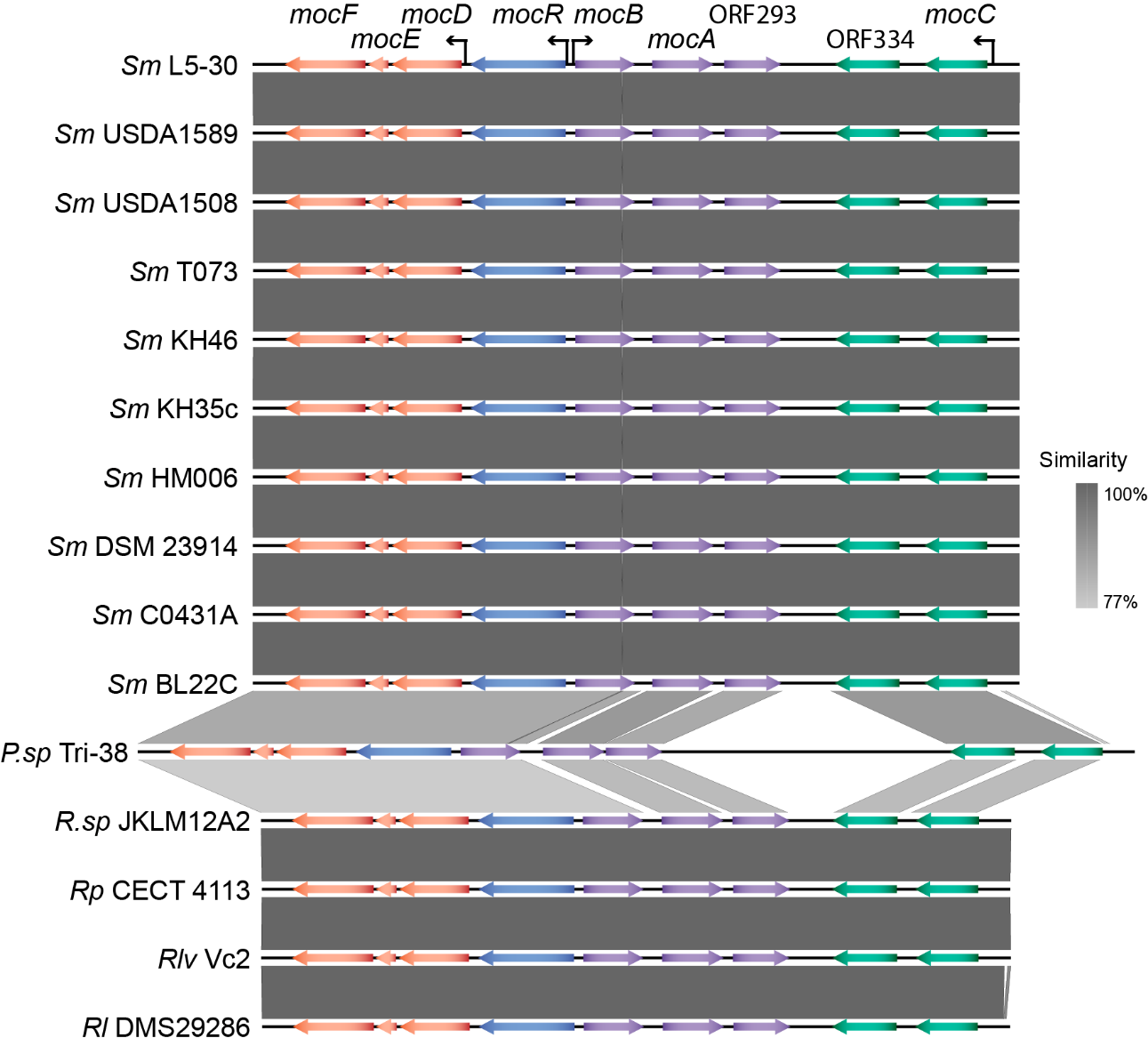
**

**Fig S1. Synteny alignment of bacteria carrying rhizopine catabolic genes.** All bacteria carrying the full suite of rhizopine catabolic genes were identified based on BLASTP analysis. A synteny alignment of the genetic regions was constructed using EasyFig (Sullivan et al., 2011). Species and genus names are as follows; *Sm Sinorhizobium meliloti; P Phyllobacterium; R. Rhizobium; Rp Rhizobium pisi; Rl Rhizobium leguminiosarum; Rlv Rhizobium leguminosarum* bv. *viciae*.

**
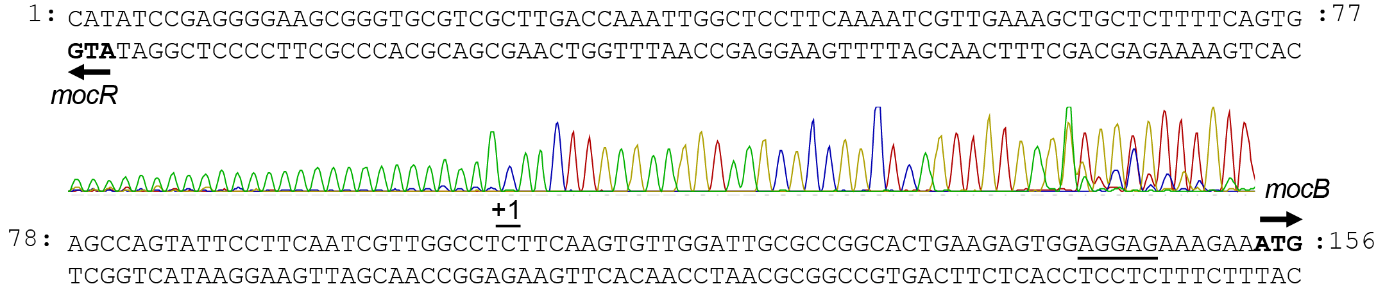
**

**Fig S2. Rapid amplification of cDNA ends (5’-RACE) of the P*mocB* promoter.** The chromatogram trace aligned to the *mocB* promoter sequence indicates the transcriptional start site is a cytosine nucleotide positioned 46-bp upstream of the *mocB* start codon.

**
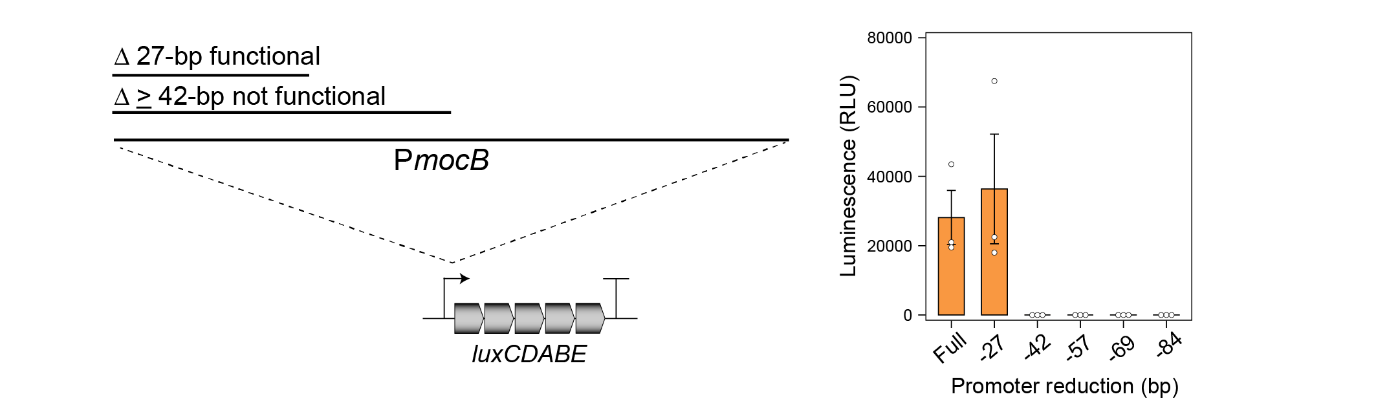
Fig S3. identification of the minimal functional P*mocB* promoter region.** A series of P*mocB* promoter regions was amplified with increments of DNA removed from the 5’-end then transcriptionally fused to a *luxCDABE* cassette in the plasmid backbone pOGG093. The plasmid was mobilised into *Rlv* 3841 carrying pSIR02b and bioluminescence was monitored in UMS cultures supplemented with 10 µM SI.

**
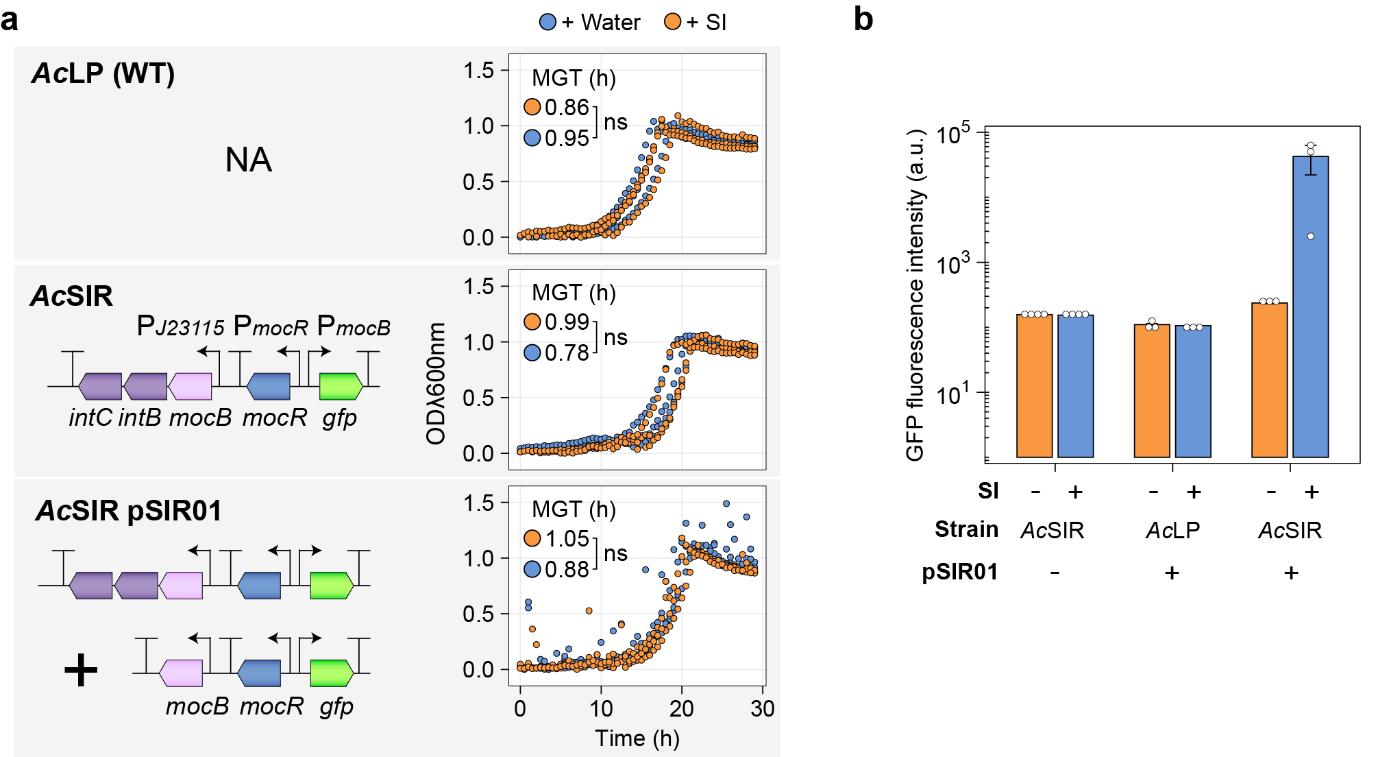
**

**Fig S4. Growth curves for *Azorhizobium caulinodans* *Ac*LP and strains carrying a single-copy mini-Tn7 integrated rhizopine biosensor derived from pSIR02.** Strains were grown in UMS media supplemented with 10 mM NH_3_Cl as a sole source of nitrogen and 20 mM Na succinate as a sole carbon source. Growth statistics including mean generation time (MGT) was calculated using the R package Growthcurver. **(b)** GFP fluorescence intensity was assessed by flow-cytometry in UMS cultures +/- 10 µM SI. Error bars represent one SEM (*n =* 3). Independent two-tailed students t-tests were used to compare means. Not significant (ns P > 0.05).
