# Supplementary material for: Rhizopine biosensors for plant‐dependent control of bacterial gene expression
Source: Environ Microbiol. 2022 Dec 4;25(2):383–96. doi: 10.1111/1462-2920.16288 (PMC10107442; doi:10.1111/1462-2920.16288)
Supplement: Supplementary file 2 — Table S1. Bacterial strains used in this study. [file EMI-25-383-s004.docx]

**Table S1. Bacterial strains used in this study**

| **Strain** | **Description** | **Reference** |
| --- | --- | --- |
| *Azoarcus olearius* DQS-4 | Betaproteobacterium, isolated from oil-contaminated soil in Taiwan. | (Chen et al., 2013) |
| *Azorhizobium caulinodans* ORS571 | Alphaproteobacterium, isolated from *Sesbania rostrata* stems. | (Dreyfus et al., 1988) |
| *A. caulinodans Ac*LP | ORS571 harboring mini-Tn*7 attB* integration site from *Rhizobium* stably integrated into the chromosome. | (Haskett et al., 2021) |
| *A. caulinodans Ac*Cherry | *Ac*LP harboring a mini-Tn*7*-Gm cassette with *mCherry* expressed from the constitutive promoter J23104 | (Haskett et al., 2021) |
| *Azospirillum brasilense* FP2 | Alphaproteobacterium, spontaneous St^R^ mutant of Sp7 which was isolated from Tropical grasses in Brazil. | (Tarrand et al., 1978) |
| *Burkholderia vietnamiensis* WPB | Betaproteobacterium, isolated from *Populus* (cottonwood). | (Doty et al., 2005) |
| *Herbaspirillum seropedicae* SmR1 | Betaproteobacterium, spontaneous St^R^ mutant of Z78 which was isolated from *Sorghum bicolor* in Brazil. | (Baldani et al., 1996) |
| *Pseudomonas fluorescence* SBW25 | Gammaproteobacterium, isolated from the rice rhizosphere in southern China. | (De Leij et al., 1995) |
| *Rhizobium leguminosarum* bv. v*iceae* 3841 | Spontaneous Sm^R^ mutant of strain *R. leguminosarum* 300, symbiont of pea | (Johnston and Beringer, 1975) |
| *Rhodobacter sphaeroides* WS8 | Alphaproteobacterium, isolated from soil in Ithica, NY. | (Clayton and Clayton, 1972) |
| *Sinorhizobium meliloti* 2011 | Alphaproteobacterium, *Medicago* symbiont | (Galibert et al., 2001) |
| *S. meliloti* CL150 | *Sm* 2011 with *pstC* and *ecfR1* corrected | (Schlüter et al., 2013) |
| *S. meliloti* RFF164 | *Sm* CL150 with markerless deletion to *rpoE2-rsiA* | (Lang et al., 2018) |
| *S. meliloti* RFF231 | *Sm* CL150 with markerless deletion to *rpoH1* and *rpoH2* | (Lang et al., 2018) |
| *S. meliloti* RFF625c | *Sm* CL150 with markerless deletions to all extracytoplasmid sigma factors and putative anti-sigma factors | (Lang et al., 2018) |
| *Escherichia coli* DH5α | Gammaproteobacterium. Laboratory cloning strain. Genotype: *fhuA2 lac(del)U169 phoA glnV44 Φ80' lacZ(del)M15 gyrA96 recA1 relA1 endA1 thi-1 hsdR17* | New England Biolabs |
| *E. coli* ST18 | Diparental conjugation strain S17 *λpirΔhemA* | (Thoma and Schobert, 2009) |

**References**

Baldani, J.I., Pot, B., Kirchhof, G., Falsen, E., Baldani, V.L., Olivares, F.L. et al. (1996) Emended description of *Herbaspirillum*; inclusion of *Pseudomonas rubrisubalbicans*, a milk plant pathogen, as *Herbaspirillum rubrisubalbicans* comb. nov.; and classification of a group of clinical isolates (EF group 1) as *Herbaspirillum* species 3. *Int J Syst Evol Microbiol* **46**: 802-810.

Chen, M.H., Sheu, S.Y., James, E.K., Young, C.C., and Chen, W.M. (2013) *Azoarcus olearius* sp. nov., a nitrogen-fixing bacterium isolated from oil-contaminated soil. *Int J Syst Evol Microbiol* **63**: 3755-3761.

Clayton, R.K., and Clayton, B.J. (1972) Relations between pigments and proteins in the photosynthetic membranes of *Rhodopseudomonas spheroides*. *Biochimica et Biophysica Acta* **283**: 492-504.

De Leij, F.A.A.M., Sutton, E.J., Whipps, J.M., Fenlon, J.S., and Lynch, J.M. (1995) Field Release of a Genetically Modified *Pseudomonas fluorescens* on Wheat: Establishment, Survival and Dissemination. *Nat Biotechnol* **13**: 1488-1492.

Doty, S.L., Dosher, M.R., Singleton, G.L., Moore, A.L., Aken, B.v., Stettler, R.F. et al. (2005) Identification of an endophytic Rhizobium in stems of *Populus*. *Symbiosis* **39**: 27-35.

Dreyfus, B., L., G.J., and Gillis, M. (1988) Characterization of *Azorhizobium caulinodans* gen. nov., sp. nov., a stem-nodulating nitrogen-fixing bacterium isolated from *Sesbania rostrata*. *Int J Syst Evol Microbiol* **38**: 89-98.

Galibert, F., Finan, T.M., Long, S.R., Pühler, A., Abola, P., Ampe, F. et al. (2001) The composite genome of the legume symbiont *Sinorhizobium meliloti*. *Science* **293**: 668-672.

Haskett, T.L., Knights, H.E., Jorrin, B., Mendes, M.D., and Poole, P.S. (2021) A simple in situ assay to assess plant-associative bacterial nitrogenase activity. *Front Microbiol* **12**.

Johnston, A.W., and Beringer, J.E. (1975) Identification of the *Rhizobium* strains in pea root nodules using genetic markers. *J Gen Microbiol* **87**: 343-350.

Lang, C., Barnett, M.J., Fisher, R.F., Smith, L.S., Diodati, M.E., and Long, S.R. (2018) Most *Sinorhizobium meliloti* extracytoplasmic function sigma factors control accessory functions. *mSphere* **3**.

Schlüter, J.-P., Reinkensmeier, J., Barnett, M.J., Lang, C., Krol, E., Giegerich, R. et al. (2013) Global mapping of transcription start sites and promoter motifs in the symbiotic α-proteobacterium *Sinorhizobium meliloti* 1021. *BMC Genomics* **14**: 156.

Tarrand, J.J., Krieg, N.R., and Döbereiner, J. (1978) A taxonomic study of the *Spirillum lipoferum* group, with descriptions of a new genus, *Azospirillum* gen. nov. and two species, *Azospirillum lipoferum* (Beijerinck) comb. nov. and *Azospirillum brasilense* sp. nov. *Can J Microbiol* **24**: 967-980.

Thoma, S., and Schobert, M. (2009) An improved *Escherichia coli* donor strain for diparental mating. *FEMS Microbiol Lett* **294**: 127-132.
