# Supplementary material for: Rhizopine biosensors for plant‐dependent control of bacterial gene expression
Source: Environ Microbiol. 2022 Dec 4;25(2):383–96. doi: 10.1111/1462-2920.16288 (PMC10107442; doi:10.1111/1462-2920.16288)
Supplement: Supplementary file 3 — Table S2. Plasmids used in this study. [file EMI-25-383-s002.docx]

**Table S2. Plasmids used in this study**

| **Plasmid** | **Replicon** | **Antibiotic resistance** | **Description** | **Ref** |
| --- | --- | --- | --- | --- |
| pBBR-MCS2 | pBBR1 | Kan | Broad-host range cloning vector | (Kovach et al., 1995) |
| pMQ131-PAR | pBBR1, CEN6 | Kan | Broad-host range cloning plasmid with yeast origin CEN6 and URA3 | Min Ryu |
| pOGG003 | ColE1 | Spec | pL0M-T Pharmacia Level 0 golden-gate terminator module | (Geddes et al., 2019a) |
| pOGG037 | ColE1 | Spec | pL0M-SC *GFP* Level 0 golden-gate SC module | (Geddes et al., 2019a) |
| pOPS0046 | RK2 | Tet | Rhizopine biosensor with P*mocB*::*luxCDABE* transcriptional fusion, no *intBC* | (Geddes et al., 2019b) |
| pOGG068 | ColE1 | Spec | pL0V-PU Level 0 golden-gate SC destination vector | (Geddes et al., 2019a) |
| pOGG072 | ColE1 | Spec | pL0V-SC Level 0 golden-gate SC destination vector | (Geddes et al., 2019a) |
| pOGG093 | RK2 | Tet | Stable broad-host-range golden-gate destination vector, very low copy number | (Geddes et al., 2019a) |
| pOGG097 | ColE1 | Spec | pL0M-PU *mocR* promoter Level 0 golden-gate SC module | This study |
| pOGG098 | ColE1 | Spec | pL0M-PU *mocB* promoter Level 0 golden-gate SC module | This study |
| pOGG109 | ColE1 | Spec | pL0M-SC *mocR* Level 0 golden-gate SC module | This study |
| pOGG121 | ColE1 | Spec | pL0M-P PJ23106 Level 0 golden-gate promoter module | (Grant, 2019) |
| pOGG143 | ColE1 | Spec | pL0M-U [RStd] Level 0 golden-gate RBS module | (Grant, 2019) |
| pOGG157 | ColE1 | Spec | pL0M-T DT16 Level 0 golden-gate terminator module | (Grant, 2019) |
| pOGG194 | ColE1 | Spec | pL0M-PU *mocC* promoter Level 0 golden-gate SC module | This study |
| pOGG195 | ColE1 | Spec | pL0M-PU *mocD* promoter Level 0 golden-gate SC module | This study |
| pOPS0365 | pBBR1 | Gent | pOGG024 carrying *intBC* from *Rlv* expressed from Plac promoter. | (Haskett et al., 2022b) |
| pOPS0755 | pBBR1 | Gent | pOGG024 with P*mocB*::GFP translational fusion | This study |
| pOPS0756 | pBBR1 | Gent | pOGG024 with P*mocC*::GFP translational fusion | This study |
| pOPS0757 | pBBR1 | Gent | pOGG024 with P*mocD*::GFP translational fusion | This study |
| pOPS1111 | pBBR1 | Gent | pOGG024 with P*mocR*::GFP translational fusion | This study |
| pOPS1112 | RK2 | Tet | pOGG093 with *mocR* expressed from P*J23106* with RBS Rstd | This study |
| pOPS1198 | RK2 | Tet | pOGG093 with P*mocB* (full)::*luxCDABE* fusion | This study |
| pOPS1204 | RK2 | Tet | pOGG093 with P*mocB* (truncated 27-bp from 5’-end)::*luxCDABE* fusion | This study |
| pOPS1205 | RK2 | Tet | pOGG093 with P*mocB* (truncated 43-bp from 5’-end)::*luxCDABE* fusion | This study |
| pOPS1206 | RK2 | Tet | pOGG093 with P*mocB* (truncated 58-bp from 5’-end)::*luxCDABE* fusion | This study |
| pOPS1207 | RK2 | Tet | pOGG093 with P*mocB* (truncated 73-bp from 5’-end)::*luxCDABE* fusion | This study |
| pOPS1208 | RK2 | Tet | pOGG093 with P*mocB* (truncated 88-bp from 5’-end)::*luxCDABE* fusion | This study |
| pOPS1740 | R6K, CEN6 | Gent, Carb | Mini-Tn7 delivery vector with rhizopine biosensor derived from pSIR02 | This study |
| pOPS1744 | R6K, CEN6 | Gent, Carb | Mini-Tn7 cloning vector withyeast origin CEN6 and URA3 marker | This study |
| pOPS1951 | pBBR1 | Gent | pOGG024 with P*rpoD*::[RStd]::GFP transcriptional fusion | This study |
| pOPS1952 | pBBR1 | Gent | pOGG024 with P*ropB1*::[RStd]::GFP transcriptional fusion | This study |
| pOPS1953 | pBBR1 | Gent | pOGG024 with P*topA*::[RStd]::GFP transcriptional fusion | This study |
| pOPS1954 | pBBR1 | Gent | pOGG024 with P*lac*::[RStd]::GFP transcriptional fusion | This study |
| pOPS1955 | pBBR1 | Gent | pOGG024 with P*empty*::[RStd]::GFP transcriptional fusion | This study |
| pOPS2003 | pBBR1 | Gent | pOGG024 with P*mocR*::[RStd]::GFP transcriptional fusion | This study |
| pSIR01 | pBBR1, CEN6 | Kan | Rhizopine biosensor with P*mocB*::*GFP* transcriptional fusion, no *intBC* | (Haskett et al., 2022b) |
| pSIR02 | pBBR1, CEN6 | Kan | Rhizopine biosensor with P*mocB*::*GFP* transcriptional fusion | (Haskett et al., 2022b) |
| pSIR02b | pBBR1 | Kan | Rhizopine biosensor with P*mocB*::*GFP* transcriptional fusion, no PAR genes | This study |
| pSIR03 | RK2 | Tc | Rhizopine biosensor with P*mocB*::*GFP* transcriptional fusion | (Haskett et al., 2022a) |
| pSIR04 | RK2 | Tc | Rhizopine biosensor (pSIR03 with GFP removed) | This study |
| pSIR05 | pBBR1, CEN6 | Kan | Rhizopine biosensor with P*mocB*::*GFP* transcriptional fusion and reduced expression of *intBC* | This study |
| pTNS3 | R6K | Carb | Tn7 transposase helper plasmid | (Choi and Schweizer, 2006) |

**References**

Choi, K.H., and Schweizer, H.P. (2006) mini-Tn7 insertion in bacteria with single attTn7 sites: example *Pseudomonas aeruginosa*. *Nat Protoc* **1**: 153-161.

Geddes, B.A., Mendoza-Suárez, M.A., and Poole, P.S. (2019a) A bacterial expression vector archive (BEVA) for flexible modular assembly of golden gate-compatible vectors. *Front Microbiol* **9**: 3345.

Geddes, B.A., Paramasivan, P., Joffrin, A., Thompson, A.L., Christensen, K., Jorrin, B. et al. (2019b) Engineering transkingdom signalling in plants to control gene expression in rhizosphere bacteria. *Nat Commun* **10**: 3430.

Grant, K. (2019) Engineering rhizobacteria as synthetic biology chassis. In *Department of Plant Sciences*: University of Oxford.

Haskett, T.L., Karunakaran, R., Bueno Batista, M., Dixon, R., and Poole, P.S. (2022a) Control of nitrogen fixation and ammonia excretion in *Azorhizobium caulinodans*. *PLoS Genet* **18**: e1010276.

Haskett, T.L., Paramasivan, P., Mendes, M.D., Green, P., Geddes, B., Knights, H.E. et al. (2022b) Engineered plant control of associative nitrogen fixation. *Proc Natl Acad Sci* **119**: e2117465119.

Kovach, M.E., Elzer, P.H., Hill, D.S., Robertson, G.T., Farris, M.A., Roop, R.M., 2nd, and Peterson, K.M. (1995) Four new derivatives of the broad-host-range cloning vector pBBR1MCS, carrying different antibiotic-resistance cassettes. *Gene* **166**: 175-176.
